# Complete Genome Sequences of Two Human Parainfluenza Virus Type 3 Isolates Collected in Brazil

**DOI:** 10.1128/genomeA.01192-17

**Published:** 2017-12-07

**Authors:** Nicholas Di Paola, Marielton dos Passos Cunha, Giuliana Stravinskas Durigon, Eitan Naaman Berezin, Edison Durigon, Danielle Bruna Leal de Oliveira, Paolo Marinho de Andrade Zanotto

**Affiliations:** a Department of Microbiology, Institute of Biomedical Sciences, University of Sao Paulo, Sao Paulo, Brazil; b Department of Pediatrics, Santa Casa de Misericórdia Hospital, Sao Paulo, Brazil; c Infectious Disease Division, Children’s Institute University of Sao Paulo, HC-FMUSP, Sao Paulo, Brazil

## Abstract

Here, we present the complete genome sequences of two human parainfluenza virus type 3 (HPIV-3) isolates collected from hospitalized infants suffering from acute respiratory disease. These are the first complete genome sequences of HPIV-3 originating from Brazil.

## GENOME ANNOUNCEMENT

Human parainfluenza viruses (HPIVs) are single-stranded RNA viruses and part of the *Paramyxoviridae* family. The four types of HPIV exist in two distinct genera, *Respirovirus* (HPIV-1 and HPIV-3) and *Rubulavirus* (HPIV-2 and HPIV-4). HPIV-3 has a higher prevalence than other HPIVs and is responsible for nearly 11% of all pediatric hospitalizations for acute respiratory infections (1, 2). In Brazil, HPIV-3 infections leading to pediatric hospitalization have been reported to be as high as 8.3% (3).

In 2010, a prospective study of acute respiratory infection surveillance was conducted in children under 24 months old. Included in this study were patients who presented at the time of their admission signs and symptoms of lower respiratory tract infection, including history of coughing and/or respiratory distress and/or those with one or more of the following clinical diagnoses: bronchiolitis, alveolar pneumonia, wheezing, bronchospasm, croup, coqueluchoide syndrome, whooping cough, cyanosis, and apnea. Sixteen patients tested negative for all PCR diagnostic assays for common respiratory viruses. To discover the etiological pathogen and improve surveillance, patient samples were processed for next-generation sequencing.

Viral RNA was extracted from the nasopharyngeal aspirates using the QIAamp viral RNA minikit (Qiagen, Valencia, CA, USA), purified with DNase I, and concentrated using the RNA Clean and Concentrator TM-5 kit (Zymo Research, Irvine, CA, USA). The paired-end RNA libraries were constructed and validated using the TruSeq Stranded Total RNA HT sample prep kit (Illumina, San Diego, CA, USA). Sequencing was done at the Core Facility for Scientific Research–University of São Paulo (CEFAP-USP/GENIAL) using the Illumina NextSeq platform. Each sample was barcoded individually, which allowed separation of reads for each patient. Short unpaired reads and bases and low-quality reads were removed using Trimmomatic version 0.36 (4). Paired-end reads (Phred quality score, >33) were assembled *de novo* with SPAdes version 3.10 using default parameters (5).

For two patients, STA762 and STA829, the largest assembled contig was identified as HPIV-3 using BLAST searches. We extracted the consensus sequences using Geneious version 9.1.2 (6). For STA762, the sequence had a length of 15,450 nucleotides (nt) with 1,415.6× average depth. Likewise, the sequence extracted from STA829 had a length of 15,422 nt with 20.2× average depth. When aligned with the HPIV-3 reference genome sequence (GenBank accession number NC_001762), both sequences exceeded a 99.8% breadth of coverage, with bases missing only at the extremes of the 5′ and 3′ untranslated regions.

A maximum-likelihood tree was estimated using FastTree version 2.1 (7), which included publicly available HPIV-3 complete genomes from GenBank. We found that our sequences were different, as they existed in distinct genotypes. STA762 was most closely related to the 9F8 strain sequence (GenBank accession number KY684748) from the United States (nucleotide pairwise identity, 99%), while the STA829 sequence exhibited a close relation to the HPIV3/MEX/1077/2004 strain sequence (GenBank accession number KF687319) from Mexico (nucleotide pairwise identity, 99%).

The reported sequences represent the first complete HPIV sequenced genomes from Brazil and suggest that there are multiple genotypes circulating in the state of Sao Paulo.

### Accession number(s).

The complete genome sequences of isolates STA762 and STA829 have been submitted to GenBank under the accession numbers MF987836 and MF987837, respectively.
